# Does Prophylactic Administration of Edoxaban Increase D-Dimer Levels after Total Hip Arthroplasty?

**DOI:** 10.3390/jcm8050678

**Published:** 2019-05-14

**Authors:** Toshiyuki Kawai, Yutaka Kuroda, Koji Goto, Shuichi Matsuda

**Affiliations:** Department of Orthopaedic Surgery, Kyoto University Graduate School of Medicine, 54 Shogoin-Kawaharacho, Sakyo-ku, Kyoto 606-8507, Japan; ykuromd@gmail.com (Y.K.); k.g.bau@kuhp.kyoto-u.ac.jp (K.G.); smat522@kuhp.kyoto-u.ac.jp (S.M.)

**Keywords:** total hip arthroplasty, D-dimer, edoxaban, deep vein thrombosis

## Abstract

This study aimed to examine the effect of thromboprophylactic edoxaban on D-dimer levels and anemia after total hip arthroplasty (THA). We retrospectively analyzed data from 349 patients undergoing primary THA. Univariate regression and multivariate regression analyses were performed with D-dimer levels on the 7th, 14th, and 21st days postoperatively as the dependent variable Edoxaban use, age, sex, body mass index (BMI), renal function, drop in hemoglobin (Hb) drop, intraoperative blood loss and duration of surgery as were independent variables. Multivariate regression analysis was also performed with Hb drop as the dependent variable. Edoxaban administration of 15 mg/day and 30 mg/day after THA was correlated with higher D-dimer levels at 21, but not at 7 or 14, days postoperatively. Other significant independent predictors for high D-dimer levels were the duration of surgery (at 7 and 14 days), BMI (at 7 days), sex (at 14 days), and age (at 14 and 21 days). Edoxaban use was significantly, weakly correlated with a larger Hb drop at 7 and 14 days, but this was less than a clinically important difference. D-dimer levels after THA can be elevated by thromboprophylactic edoxaban after three weeks postoperatively.

## 1. Introduction

Patients who undergo total hip arthroplasty (THA) are at high risk of postoperative venous thromboembolism (VTE). Without any prophylaxis, the incidence of symptomatic or asymptomatic deep vein thrombosis (DVT) is more than 40% [1].

A recent systematic review showed that new oral anticoagulants, such as rivaroxaban, dabigatran, and apixaban, are cost-effective alternatives to low-molecular-weight heparin to prevent VTE after THA and total knee arthroplasty (TKA) [2]. Edoxaban is an oral direct factor Xa inhibitor for the prevention and treatment of DVT [3]. Previous studies involving patients who underwent THA showed that oral edoxaban was safe and effective for preventing VTE after total hip arthroplasty [4,5]. However, there is still concern regarding local bleeding associated with anticoagulant administration after major surgery. With the possible exception of apixaban, newer anticoagulants that lower the risk of postoperative VTE increase bleeding [6]. Administration of 60 mg/day edoxaban resulted in a larger hemoglobin (Hb) drop than 10 mg/day apixaban administration for treatment of DVT after TKA or THA [7]. Prophylactic administration of 30 mg edoxaban after THA is associated with more anemia than administration of 2.5 mg fondaparinux [8]. However, Fuji et al reported that oral edoxaban 30 mg once daily was superior to subcutaneous enoxaparin 2000 IU twice daily for preventing VTE following THA without increasing the risk for major or clinically relevant non-major bleeding [9].

The current standard diagnostic approach of DVT and pulmonary embolism (PE) relies on sequential diagnostic tests, including plasma D-dimer measurement, ultrasonography, or computed tomography. D-dimer measurement is widely performed for excluding DVT and PE because of its high sensitivity and simplicity [10,11].

Several researchers have attempted to define an optimal D-dimer cutoff level to detect DVT and PE by adjusting to age [12,13,14]. However, D-dimer levels can be affected by acute phase reactions after surgery, and knowing which factor affects D-dimer levels could be important.

D-dimer is a product of fibrin degradation, which reflects coagulation and fibrinolytic processes that occur after surgery [15]. These processes might correlate with the degree of residual local bleeding at the surgical site. If anticoagulant agents delay hemostasis, this could result in a higher amount of local hemorrhage and prolonged process of fibrin degradation. This potentially elevates D-dimer levels and delays a reduction in D-dimer levels after surgery.

Several reports have compared the effect of edoxaban on D-dimer levels and the degree of anemia after total arthroplasty surgery [7,8,9,16]. However, no studies have evaluated the effect of edoxaban on D-dimer levels compared with no anticoagulant control after THA. The primary goal of this study was to examine if the use of edoxaban after THA affects D-dimer levels. Effects of edoxaban on a reduction in Hb after surgery were also examined. We also aimed to determine which factors affect D-dimer levels and a reduction in Hb after THA.

## 2. Patients and Methods

**Figure 1:**
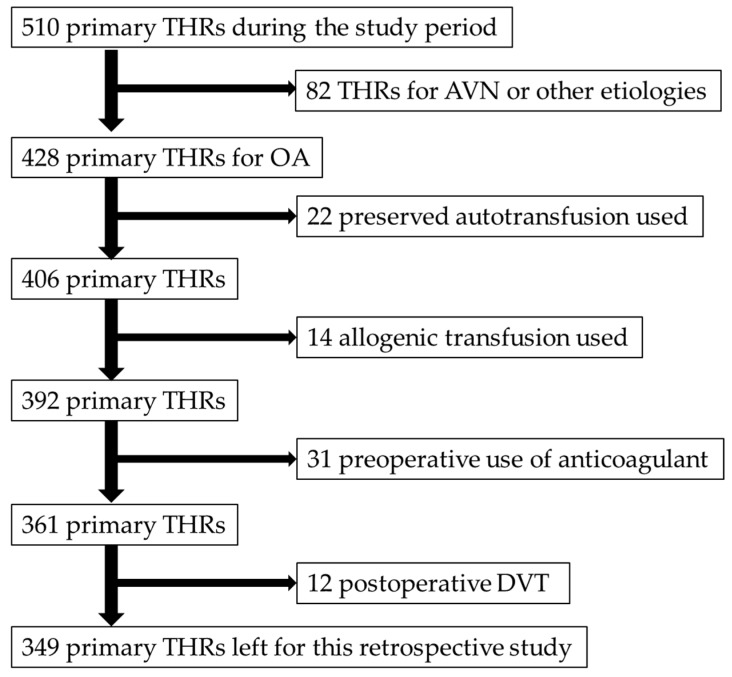
Patients inclusion and exclusion algorithm. THA: total hip arthroplasty, AVN: avascular necrosis, OA: osteoarthritis, DVT: deep vein thrombosis.

This was a retrospective study for primary THAs performed from April 2012–March 2017. All patients provided informed consent and the study protocol was approved by the Institutional Review Board of our hospital. In total, 510 primary THAs were performed at our institute during the study period, and exclusions and remaining number of patients in the study are shown in Figure 1. Eighty-two THAs that were performed for reasons other than osteoarthritis, such as avascular necrosis of the femoral head, were excluded from this study. Twenty-two procedures were excluded because of using preserved autotransfusion that was prepared a couple of weeks before surgery. Autotransfusion was performed on the day of index surgery, which could potentially affect D-dimer and Hb values after surgery. Another 14 procedures were excluded because of allogenic transfusion after surgery. One or more anticoagulants were administered preoperatively for reasons such as cardiovascular and cerebral infarction. These anticoagulants were continued postoperatively for 31 patients, who were also excluded from this study. Postoperative DVT was confirmed in 12 patients, all of whom were asymptomatic and eliminated from this study. Since the presence of DVT would largely affect the D-dimer level, the effect of edoxaban and that of DVT should be examined independently; however, we felt that the number of DVT cases (12 patients) were too small to be examined independently or to be included as a candidate factor for multivariate analysis. A total of 349 primary THAs remained.

The average age at the time of surgery was 62.4 years (17–87 years) and the average BMI was 23.8 (13.2–41.8) kg/m^2^. All 349 procedures were performed by a senior hip surgeon (K.G., Y.K., K.S. or H.A.). All surgeries were performed through the anterolateral approach or the minimally invasive anterolateral approach [17]. Edoxaban was administered postoperatively for 121 (34.7%) patients as a prophylaxis for DVT, of which edoxaban 15 mg/day was used for 43 (group E15) patients and 30 mg/day was used for 78 (group E30) patients. No thromboprophylaxis was administered preoperatively or postoperatively for the remaining 228 (65.3%) patients (group N). The prophylaxis strategy was based on the Japanese guidelines 2004 that recommended consideration of either anticoagulant therapy or intermittent pneumatic compression for patients undergoing THA [18]. An intermittent pneumatic compression device was used for all three groups. A prophylactic anticoagulant was administered for relatively inactive patients, but the decision on whether to administer edoxaban mainly relied on the surgeon’s preference. The choice of the dose (15 mg or 30 mg) was subjected to a chronological change of strategy made by the pharmacist in charge of the Orthopedic Department, not based on the patient’s body weight or renal function. Edoxaban 15 mg/day was mainly used until March 2014 and 30 mg/day was used since April 2014. The demographic data for each group are shown in Table 1.

Treatment with the anticoagulant drug was initiated in the morning two days after surgery and then continued for 14 days. A blood sample was taken at 7 am on the 1st, 7th, 14th, and 21st postoperative days, and included Hb and D-dimer measurement. Plasma D-dimer levels were measured using a latex agglutination kit (LSIA GENESIS D-dimer; LSI Medience Corporation, Tokyo, Japan) with 1.0 μg/mL as the positive threshold. The blood test at these time points was not set up for this study, but had been taken as routine prior to this study period. The results of the blood test on postoperative day 1 were not used for analysis because results on the 1^st^ day can be largely affected by what time the surgery was on the previous day. The timing of blood sampling was fixed because fibrinolytic activity changes with the time of day [19].

All patients received mechanical prophylaxis for DVT consisting of intermittent pneumatic compression, compression stockings, and early ambulation, which started on postoperative day 2 with full weight bearing. When clinical symptoms suggestive of DVT were observed or a D-dimer level was more than 20 μg/mL, an ultrasound examination was performed to detect thrombosis. When thrombosis was found, an anticoagulant drug was initiated or added after consultation with cardiologists. As mentioned above, DVT was detected in 12 of 361 patients and those patients were excluded from analysis.

We considered the following covariates: sex, body mass index (BMI), age, creatinine level, estimated glomerular filtration rate (eGFR), Hb change after surgery, intraoperative blood loss, and duration of surgery. Creatinine and eGFR were included because several studies showed a relationship between renal function and the coagulation system [20,21,22,23]. Because edoxaban possibly affects a drop in Hb after THA, the effect of the above-mentioned factors on a drop in Hb was also examined.

### Statistical Analysis

Differences in proportions were calculated by the chi-square test. Differences in means were calculated by the Wilcoxon test to compare two groups, or by the Kruskal–Wallis test followed by the post-hoc Steel–Dwass test to compare more than two groups. Probability values <0.05 were considered significant. Univariate regression using the D-dimer value at each time point as a dependent variable was performed when the independent variable was a continuous variable. Multivariate regression analysis was performed using the stepwise regression model with D-dimer at each time point as the dependent variable. Use of edoxaban (none or either of 15 mg/day or 30 mg/day), age, sex, BMI, change in Hb compared with the preoperative examination, surgery time, and intraoperative blood loss were independent variables. To define factors that affect Hb drop after THA, multivariate regression analysis was also performed using the change in Hb from preoperatively to each time point as the dependent variable. Use of edoxaban (none or either of 15 mg/day or 30mg/day), age, sex, BMI, surgery time, and intraoperative blood loss were independent variables.

All statistical analyses were performed using JMP Pro 14 software (SAS Institute, Cary, NC, USA).

## 3. Results

All the data are shown as mean (standard deviation (SD); range). For all of the 349 patients, mean Hb was 13.0 (SD:1.5; range: 9.4–19.0) g/dL before surgery, 11.1(1.5; 7.7–15.5) g/dL 1 day after surgery, 10.5 (1.3; 7.2–13.7) g/dL 1 week after surgery, 10.7 (1.7; 7.6–14.6) g/dL 2 weeks after surgery, and 11.0 (4.8; 8.2–14.9) g/dL 3 weeks after surgery. D-dimer values were 1.2 (1.2; 0.2 to 9.1) μg/mL before surgery, 6.5 (9.0; 0.7–40.7) μg/mL 1 day after surgery, 8.7 (4.7; 2.2–34.9) μg/mL 1 week after surgery, 9.0 (5.4; 1.3–33.7) μg/mL 2 weeks after surgery, and 7.2 (4.4; 1.2–19.5) μg/mL 3 weeks after surgery. Demographics and postoperative Hb and D-dimer levels for each anticoagulant group are shown in Table 1. The proportion of males/females, age, BMI, preoperative creatinine levels, and eGFR were not significantly different among the three groups. The mean age in group E15 was slightly higher than that for group N and group E30; however, there was no statistical difference between groups E15 and N (*p* = 0.058) or between groups E15 and E30 (*p* = 0.089). Preoperative Hb levels in group E15 were significantly lower than those in groups N and E30 (*p* = 0.0189 and *p* = 0.0161, respectively). Patients in group E15 tended to have a smaller Hb drop from preoperatively to postoperative day 14 than did those in group E30 (*p* = 0.069).

D-dimer levels at postoperative day 7 were measured in 349 (100%) patients. A total of 228 patients were in group N, 43 were in group E15, and 78 in group E30. At postoperative 14, day D-dimer levels were measured in 339 (97.1%) patients (227 in group N, 42 in group E15, and 70 in group E30). D-dimer levels at postoperative day 21 were measured in 199 (57.0%) patients (127 in group N, 37 in group E15, and 35 in group E30).

**Figure 2:**
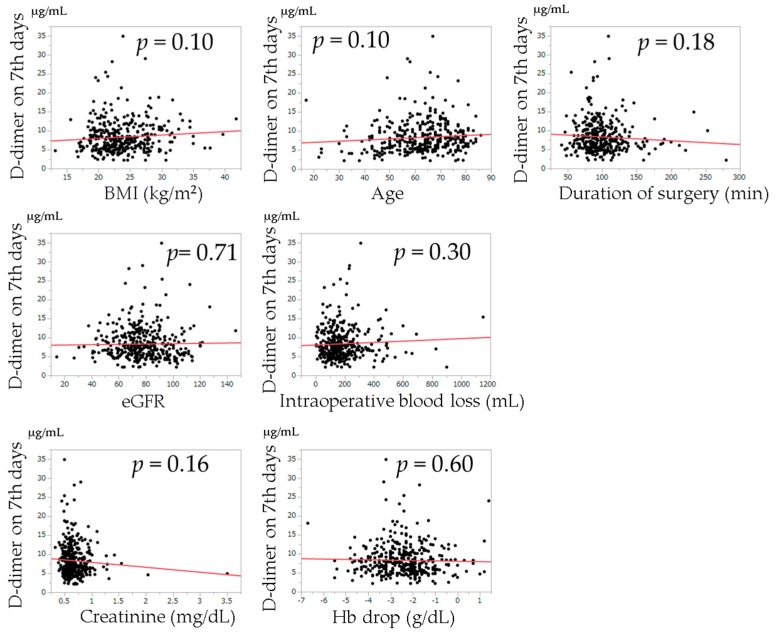
Univariate regression analysis of the relationships between each variable and D-dimer levels on the 7th postoperative day. The solid lines indicate regression lines. BMI: body mass index, eGFR: estimated glomerular filtration rate.

**Figure 3:**
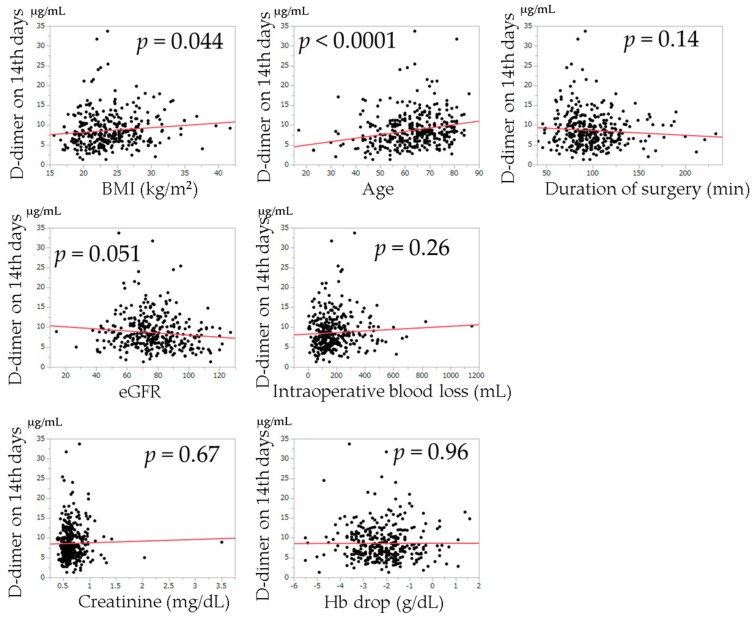
Univariate regression analysis of the relationships between each variable and D-dimer levels on the 14th postoperative day. The solid lines indicate regression lines.

**Figure 4:**
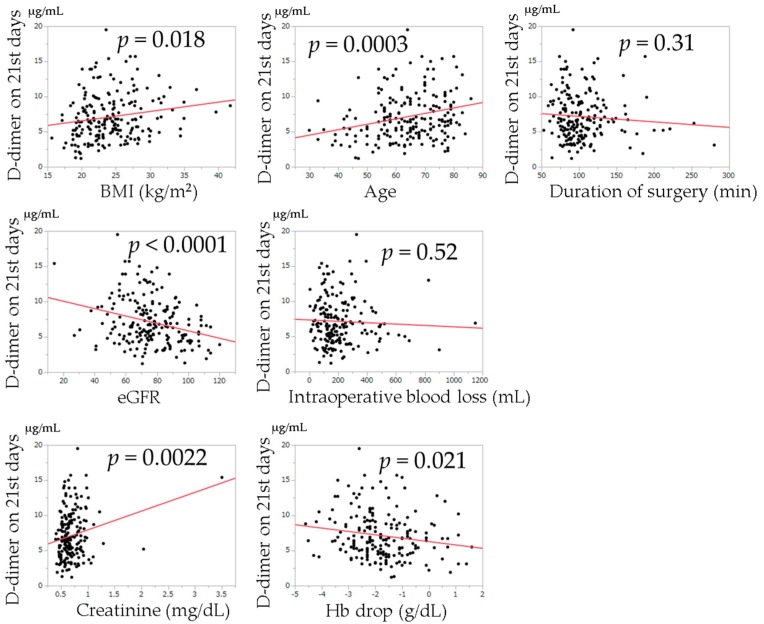
Univariate regression analysis of the relationships between each variable and D-dimer levels on the 21st postoperative day. The solid lines indicate regression lines.

The relationships between postoperative D-dimer levels and age, BMI, duration of surgery, intraoperative blood loss, change in Hb from the preoperative examination, creatinine value, and eGFR examined by univariate regression analysis are shown in Figure 2, Figure 3 and Figure 4. Univariate regression analysis showed that no factors were significantly associated with D-dimer values at postoperative day 7. Higher D-dimer levels at postoperative day 14 were significantly correlated with an older age (*p* < 0.0001). Higher D-dimer levels at postoperative day 21 were significantly correlated with a higher BMI (*p* = 0.018), higher age (*p* = 0.0003), higher creatinine level (*p* = 0.0022), lower eGFR (*p* < 0.0001), and change in Hb (*p* = 0.021).

In the multivariate model, higher D-dimer values at postoperative day 7 were significantly correlated with a higher BMI (OR: 1.04, 95%CI: 1.01–1.07, *p* = 0.015) and duration of surgery (OR: 0.996, 95%CI: 0.991–0.999, *p* = 0.044) (Table 2). Higher D-dimer values at postoperative day 14 were significantly correlated with older age (OR: 0.996, 95%CI: 1.01–1.049, *p* < 0.001), sex (OR: 1.74, 95%CI: 1.20–2.52, *p* = 0.0032), and duration of surgery (OR: 0.994, 95%CI: 0.989–0.999, *p* = 0.018). Higher D-dimer levels at postoperative day 21 were significantly correlated with use of edoxaban use (OR: 1.49, 95%CI: 1.06–2.06, *p* = 0.020), and age (OR: 1.03, 95%CI: 1.01–1.04, *p* = 0.0011). Use of edoxaban was not correlated with D-dimer levels at postoperative days 7 or 14 (OR: 0.84, 95%CI: 0.66–1.07, *p* = 0.15 and OR: 0.96, 95%CI: 0.74–1.25, *p* = 0.51, respectively).

Multivariate regression analysis, which was performed using the change in Hb from preoperatively to each time point as the dependent variable, and use of edoxaban (none or either of 15 mg/day or 30 mg/day), age, sex, BMI, surgery time, and intraoperative blood loss as independent variables, showed that male patients had a significantly larger Hb drop at any time point (Table 3). The eGFR was also associated with a larger Hb drop on postoperative day 14 (OR: 0.987, 95%CI: 0.975–0.999, *p* = 0.034). Intraoperative blood loss was correlated with a Hb drop on postoperative day 7 (OR: 1.0013, 95%CI: 1.0002–1.0032, *p* = 0.018), but a significant correlation was not observed at 14 or 21 days after THA (OR: 1.004, 95%CI: 0.994–1.001, *p* = 0.44 and OR: 0.999, 95%CI: 0.998–1.001, *p* = 0.95, respectively). The Edoxaban-use group showed a significantly larger Hb drop on postoperative days 7 (OR: 1.43, 95%CI: 1.12–1.84, *p* = 0.0036) and 14 (OR: 1.29, 95%CI: 1.01–1.67, *p* = 0.042), whereas this reduction was not significant on postoperative day 21 compared with group N (OR: 1.16, 95%CI: 0.82–1.34, *p* = 0.42).

## 4. Discussion

This study compared thromboprophylactic use of edoxaban with no thromboprophylactic use regarding D-dimer levels and a drop in Hb in patients who underwent THA. Use of edoxaban (15 and 30 mg/day) was correlated with a high D-dimer level on postoperative day 21, but not on postoperative days 7 and 14. Edoxaban use was also associated with a larger Hb drop on postoperative days 7 and 14 compared with no thromboprophylactic group, but this correlation disappeared by postoperative day 21. Possible explanation of this results would be the anticoagulant effect of edoxaban. As D-dimer is a product of fibrin degradation, which reflects coagulation and fibrinolytic processes that occur after surgery [15], these processes could be associated with the degree of residual local bleeding at the surgical site. If hemostasis is delayed by anticoagulant agents, this may result in a higher amount of local hemorrhage and prolonged process of fibrin degradation. This could explain the delayed reduction in D-dimer levels in edoxaban groups after surgery.

Older age is associated with high D-dimer levels [24,25]. In our study, multivariate regression analysis showed that older patients had higher D-dimer levels than did younger patients at 14, and 21 days after THA.

Higher BMI was an independent factor associated with higher D-dimer levels only at 7 days. However, there was no significant correlation between BMI and D-dimer levels at 14 or 21 days postoperatively. To the best of our knowledge, no reports have demonstrated an association between BMI and D-dimer levels after surgery, although a high BMI is a risk factor for DVT and PE.

Preoperative creatinine levels and eGFR was not correlated with D-dimer levels at any time point in this study. This finding is not consistent with a previous report that showed a significant correlation between eGFR and D-dimer levels [26]. However, the eGFR was an independent predictor of a large Hb drop after THA in this study. This finding implies that renal function affects coagulation and fibrinolysis.

In our study, patients with larger intraoperative blood loss had relatively higher D-dimer levels early after THA, but this correlation was not significant. The duration of surgery was significantly correlated with high D-dimer levels at 7 and 14 days. Use of edoxaban (15 or 30 mg daily for 14 days) was correlated with higher D-dimer levels on postoperative day 21, while there was no correlation on postoperative days 7 and 14 after THA.

Multivariate analysis showed that use of edoxaban use (15 or 30 mg/day) was significantly correlated with a higher Hb drop on postoperative days 7 and 14, but this correlation was weak (OR: 1.43, 95%CI: 1.12–1.84 on day 7 and OR: 1.29, 95%CI: 1.01–1.67) on day 14. Furthermore, the difference in Hb between groups was 0.1–0.6 g/dL, which is much less than a clinically important difference in Hb of 1.0–2.0 g/dL [27,28]. This finding is consistent with some previous reports, which showed that edoxaban administration after THA resulted in more anemia [7,8]. However, another study showed that prophylactic edoxaban administration after TKA did not increase the incidence of major or clinically relevant non-major bleeding [29].

This study has several limitations. First, this was a retrospective study where patients were not randomized to each anticoagulant prophylaxis group. Second, the use of edoxaban for preventing DVT was the surgeon’s preference. Edoxaban was used for less-active patients during the study period, but the criteria were subjective and the indication of edoxaban administration was strictly discussed among surgeons before or during the study period. Therefore, the E15 group had relatively older patients compared with the N group and E30 group (no significant difference among the groups). However, the effect of age was adjusted by multivariate analysis, which showed that the use of edoxaban was an independent predictor for high D-dimer levels on postoperative day 21. Third, patients with postoperative DVT were excluded from this study. Patients with DVT possibly might have higher D-dimer levels, which could have been decreased with the prophylactic effect of edoxaban. Obtained data in this study have a lot of implications; however, due to those limitations, further research including randomized controlled trial with a large number of patients would be necessary to emphasize the results in this study. Another limitation is that, although fibrinolytic activity varies with the season [30], we included procedures that were performed in any season.

## 5. Conclusions

This study shows that the prophylactic use of edoxaban 15 mg/day and 30 mg/day after THA is correlated with higher D-dimer levels on postoperative day 21, but not on postoperative days 7 and 14. Edoxaban use (15 mg/day or 30 mg/day) is weekly, but significantly correlated with a larger Hb drop until two weeks after surgery. This decrease is not a clinically important difference. Other independent predictors of high D-dimer levels are duration of surgery (significant for D-dimer levels at 7 and 14 days), BMI (at 7 days), sex (at 14 days), and preoperative creatinine levels (at 21 days).

## Figures and Tables

**Table 1:** Demographics and blood test results for each group.

Variables		Group N	Group E15	Group E30	*p* Value
N		228	43	78	
Sex	male	48	7	19	>0.05
	female	180	36	59	
BMI (kg/m^2^)		23.6 (3.9;13.2–41.2)	23.5 (4.4; 17.1–39.7)	24.0 (4.1; 16.6–36.8)	>0.05
Creatinine (mg/dL)		0.67 (0.19; 0.39–3.50)	0.65 (0.15; 0.40–1.06)	0.65 (0.16; 0.33–1.55)	>0.05
eGFR		77.6 (17.4; 14.3–120.2)	76.5 (17.0; 40.8–111.5)	80.8 (20.2; 34.4–146.2)	>0.05
Age		61.9 (11.2; 22–84)	66.6 (10.9; 35–86)	61.1 (13.5; 17–84)	>0.05
Hb (g/dL)	preoperative	13.1 (1.4; 9.4–19.0)	12.5 (1.1; 10.4–15.4)	13.2 (1.5; 9.5–16.1)	N vs E15: 0.0023 N vs E30: 0.67 E15 vs E30: 0.015
	POD7	10.7 (1.2; 7.2–13.6)	10.1 (1.4; 7.5–12.7)	10.5 (1.3; 7.7–13.7)	N vs E15: 0.0023 N vs E30: 0.87 E15 vs E30: 0.12
	POD14	10.9 (1.2; 7.6–14.6)	10.7 (1.4; 8.0–13.3)	10.8 (1.3; 7.9–13.2)	>0.05
	POD21	11.2 (1.3; 8.2–14.9)	11.1 (1.4; 8.5–14.4)	11.1 (1.4; 8.3–14.3)	>0.05
Hb Change (g/dL)	7-pre	−2.4 (1.1; −5.5–1.4)	−2.4 (1.4; −5.5–1.2)	−2.6 (1.2; −6.7–1.2)	>0.05
	14-pre	−2.1 (1.1; −5.5–1.6)	−1.8 (1.3; −5.5–0.7)	−2.4 (1.2; −5.4–1.1)	N vs E15: 0.13 N vs E30: 0.11 E15 vs E30: 0.018
	21-pre	−1.8 (1.1; −4.3–1.6)	−1.5 (1.1; −3.5–0.7)	−1.9 (1.4; −4.6–1.1)	>0.05
D-dimer (μg/mL)	POD7	8.58 (4.39; 2.2–34.9)	7.99 (4.69; 2.2–29.0)	7.76 (3.03; 2.2–18.1)	>0.05
	POD14	8.70 (4.28; 1.3–31.7)	9.02 (3.62; 2.7–17.9)	8.01 (4.45; 1.3–33.7)	>0.05
	POD21	6.73 (3.01; 1.2–15.7	7.81 (3.88; 1.3–15.7)	7.59 (3.63; 2.7–19.5)	>0.05
Intraoperative Blood Loss (mL)		183.9 (139.1; 5–1150)	222.0 (121.7; 40–520)	170.2 (149.1; 5–900)	N vs E15: 0.030 N vs E30: 0.23 E15 vs E30: 0.0050
Duration of Surgery (minute)		97.3 (25.7; 46–233)	109.3 (30.1; 64–200)	97.4 (40.6; 41–280)	N vs E15: 0.011 N vs E30: 0.41 E15 vs E30: 0.021

Data are shown as mean (standard deviation; range). BMI: body mass index, eGFR: estimated glomerular filtration rate, POD: postoperative day.

**Table 2:** Correlation coefficients and p values for each variable obtained by multivariate regression analysis with D-dimer levels as the dependent variable.

Variables	D-Dimer at 7 Days		D-Dimer at 14 Days		D-dimer at 21 Days	
	OR (95% CI)	*p* Value	OR (95% CI)	*p* Value	OR (95% CI)	*p* Value
Edoxaban use	0.84 (0.66–1.07)	0.15	0.96 (0.74–1.25)	0.51	1.49 (1.06–2.06)	0.020
Sex (male)	1.00 (0.67–1.47)	0.99	1.74 (1.20–2.52)	0.0032	1.50 (0.09–2.44)	0.078
BMI (kg/m^2^)	1.04 (1.01–1.07)	0.015	1.02 (0.99–1.05)	0.11	1.02 (0.98–1.07)	0.27
Age	1.01 (0.99–1.02)	0.070	1.02 (1.01–1.04)	<0.001	1.03 (1.01–1.04)	0.0011
Creatinine (mg/dL)	0.63 (0.32–2.00)	0.32	0.63 (0.36–1.56)	0.27	1.09 (0.54–2.91)	0.88
eGFR	1.00 (0.99–1.01)	0.67	0.99 (0.9–1.01)	0.48	0.98 (0.97–1.004)	0.12
Hgb Change from Preoperative to Follow-up Time (g/dL)	0.96 (0.88–1.06)	0.43	1.09 (0.98–1.21)	0.22	0.89 (0.77–1.05)	0.11
Intraoperative Blood Loss (mL)	1.01 (0.99–1.01)	0.12	1.001 (0.999–1.002)	0.071	1.0004 (0.999–1.002)	0.68
Duration of Surgery (minute)	0.996 (0.991–0.999)	0.044	0.994 (0.989–0.999)	0.018	0.995 (0.989–1.001)	0.25

BMI: body mass index, eGFR: estimated glomerular filtration rate, OR: Odds ratio, 95%CI: 95% confidence interval.

**Table 3:** Correlation coefficients and p values for each variable obtained by multivariate regression analysis with a drop in Hb from preoperatively to each time point as the dependent variable.

Variables	Hb Drop to 7th Day		Hb Drop to 14th Day		Hb Drop to 21st Day	
	OR (95% CI)	*p* Value	OR (95% CI)	*p* Value	OR (95% CI)	*p* Value
Edoxaban Use	1.43 (1.12–1.84)	0.0036	1.29 (1.01–1.67)	0.042	1.16 (0.82–1.34)	0.42
Sex (male)	2.07 (1.36–3.09)	<0.001	2.20 (1.43–3.33)	<0.001	2.24 (1.34–3.77)	0.0024
BMI	0.99 (0.96–1.03)	0.72	1.001 (0.970–1.034)	0.94	0.996 (1.004–1.039)	0.84
Age	1.002 (0.991–1.014)	0.72	0.99 (0.98–1.01)	0.83	1.002 (0.985–1.019)	0.83
Creatinine (mg/dL)	0.79 (0.52–2.31)	0.67	0.51 (0.17–1.49)	0.22	0.34 (0.14–1.36)	0.11
eGFR	0.998 (0.987–1.002)	0.66	0.98 (0.97–0.99)	0.034	0.99 (0.97–1.01)	0.059
Intraoperative Blood Loss (mL)	1.0013 (1.0002–1.0032)	0.018	1.004 (0.994–1.001)	0.44	0.999 (0.998–1.001)	0.95
Duration of Surgery (minute)	0.998 (0.994–1.003)	0.53	0.998 (0.993–1.004)	0.58	0.99 (0.98–1.01)	0.17

BMI: body mass index, eGFR: estimated glomerular filtration rate, OR: Odds ratio, 95%CI: 95% confidence interval.
